# A Delicate Balance: Anesthetic Management for Neonatal Congenital Diaphragmatic Hernia Repair

**DOI:** 10.7759/cureus.75506

**Published:** 2024-12-10

**Authors:** Mohamad A Omar, Tamer Ghoneim, Hind Khaleel

**Affiliations:** 1 Anesthesiology, Emirates Health Services, Sharjah, ARE

**Keywords:** congenital diaphragmatic hernia (cdh), erector spinae block (esb), high-frequency oscillatory ventilation (hfov), neonatal anesthesia, pulmonary hypertension

## Abstract

Congenital diaphragmatic hernia (CDH) presents significant challenges in neonatal management, particularly in the context of anesthesia. This case report details the successful anesthetic management of a five-day-old neonate with left-sided CDH requiring thoracoscopic repair.

A five-day-old neonate, delivered via emergency cesarean section due to breech presentation, presented with severe respiratory distress and was diagnosed with left-sided CDH. Initial evaluation revealed respiratory acidosis and moderate pulmonary hypertension. Preoperative stabilization included high-frequency oscillatory ventilation and intravenous infusions of milrinone and vasopressin. Induction was achieved with ketamine and midazolam, along with a left-sided erector spinae block for analgesia. Anesthesia was maintained with intermittent fentanyl boluses during the eight-hour surgery. Ventilation was closely monitored and adjusted for changes in carbon dioxide levels. The surgery was successful, with stable hemodynamics and a smooth recovery.

Effective anesthesia in neonates with CDH requires careful preoperative planning and intraoperative vigilance. This case underscores the importance of tailored anesthetic strategies to enhance safety and promote recovery in high-risk pediatric surgeries.

## Introduction

Congenital diaphragmatic hernia (CDH) is a life-threatening condition characterized by the abnormal development of the diaphragm, allowing abdominal contents to enter the thoracic cavity, thereby compromising pulmonary development and function. The incidence of CDH is approximately 1 in 2,500 live births, with a higher prevalence on the left side [1]. Early diagnosis and intervention are crucial, as CDH is often associated with significant respiratory distress and cardiovascular instability [2].

Anesthetic management for neonates with CDH presents unique challenges due to their compromised respiratory physiology and potential for rapid hemodynamic changes. The administration of anesthetic agents must be carefully tailored to minimize the risk of further respiratory compromise and to ensure stable hemodynamics throughout the surgical procedure [3].

This case report describes the anesthetic management of a five-day-old neonate with left-sided CDH undergoing thoracoscopic repair, highlighting the complexities involved and the strategies employed to optimize the surgical outcome.

## Case presentation

This is a five-day-old neonate delivered via emergency cesarean section at 44 weeks gestation due to breech presentation. The mother, a 41-year-old Gravida 2 Para 1 with no antenatal follow-up and a history of one previous cesarean section, presented to the emergency room with abdominal pain, leakage of amniotic fluid for 24 hours, and decreased fetal movement. There is no significant family history of genetic disorders, congenital anomalies, or prior miscarriages. It is also noted that the parents are not relatives.

**Figure 1:**
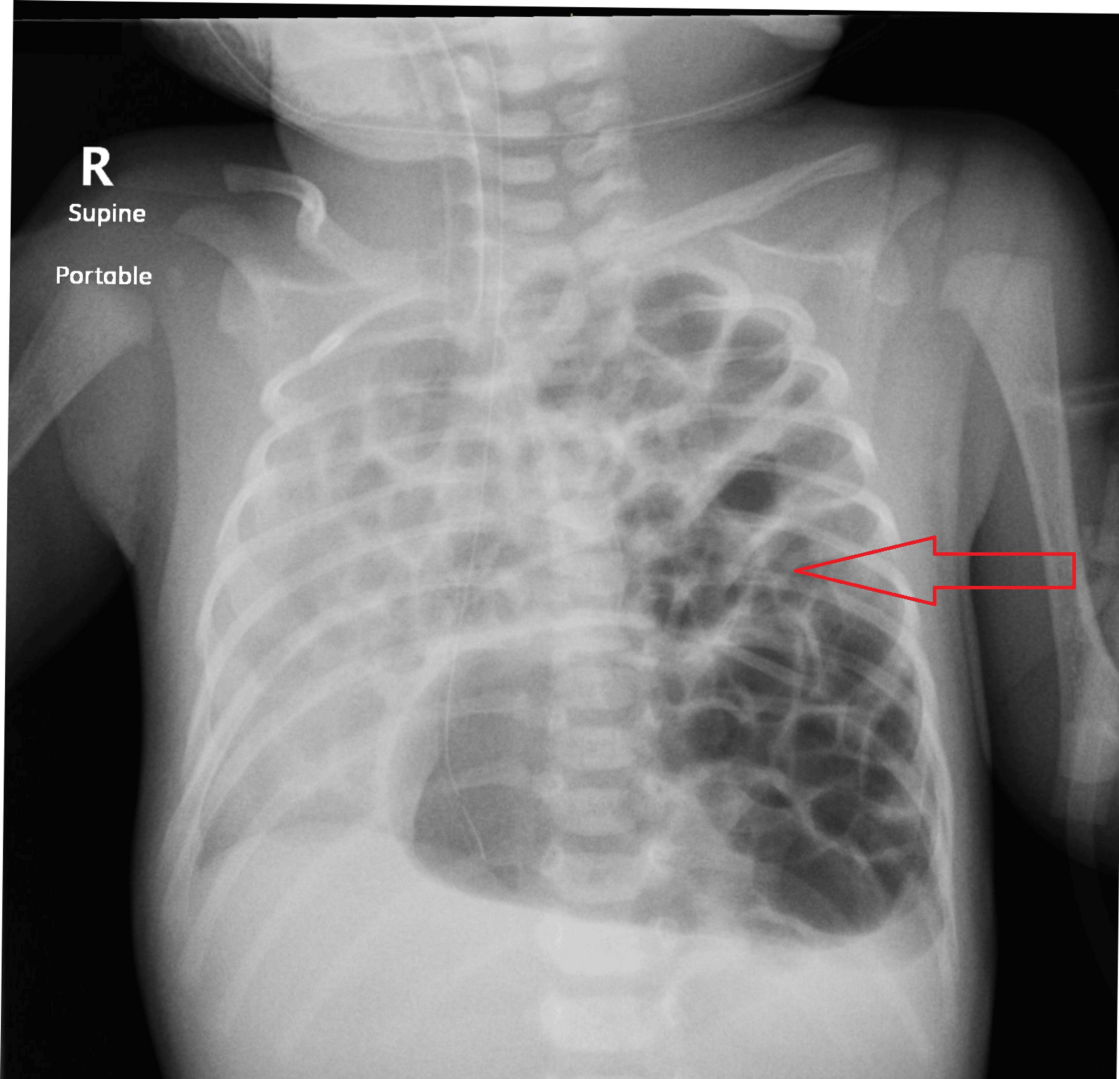
Postnatal chest X-ray taken post-delivery, demonstrating bowel loops herniating into the left hemithorax (arrow), with right mediastinal shift.

Upon delivery, the neonate was observed to be non-crying and hypoactive. The initial physical examination revealed an APGAR score of 1 at one minute and 6 at five minutes, alongside diminished air entry on the left side and a scaphoid abdomen, necessitating immediate intubation. The infant weighed 2.98 kg. Capillary blood gas analysis indicated severe respiratory acidosis, with a pH of 7.13, PCO2 of 73.7 mmHg, and HCO3 of 24.4. A chest X-ray confirmed a left diaphragmatic hernia with a right mediastinal shift as shown in Figure 1.

Subsequently, the baby was admitted to the neonatal intensive care unit (NICU) for further management. Upon admission to the NICU, an echocardiogram was performed, revealing a CDH, with the heart and aortic arch displaced to the right side. The right-sided chambers were dilated, while the left side was compressed. Moderate pulmonary hypertension was noted, indicated by a pulmonary artery systolic pressure of 46 mmHg, as well as a large patent ductus arteriosus (PDA) with a bidirectional shunt. The neonate was started on intravenous infusion and multiple medications to support hemodynamics. On day 1 post-admission, an epinephrine infusion was initiated at a dose of 0.2 mcg/kg/min to enhance cardiovascular function; this was discontinued on the second day due to improved status. Subsequently, milrinone was started to manage pulmonary hypertension at a dose of 0.3 mcg/kg/min, in conjunction with a vasopressin infusion at a dose of 0.00034 units/kg/min.

On the day of surgery, the patient was re-evaluated by the anesthesia team as part of the preoperative preparation. At that time, the patient was receiving vasopressin at a rate of 0.00034 units/kg/min and milrinone at 0.25 mcg/kg/min. Pre-ductal and post-ductal oxygen saturation levels were recorded at 91% and 78%, respectively, while the patient was on pressure-controlled high-frequency oscillatory ventilation (HFOV), set to a FiO2 of 45%, mean airway pressure of 12 cm H2O, amplitude of 25 cm H2O, frequency of 10 Hz, and an inspiratory time ratio of 1:2. Arterial blood gas analysis from the umbilical artery indicated a pH of 7.43, PCO2 of 43.2 mmHg, PO2 of 42.1 mmHg, and HCO3 of 28.6 mmol/L. Urine output remained within normal limits. A repeat echocardiogram performed on the day of surgery revealed a dilated right atrium and right ventricle with mild tricuspid regurgitation of 45 mmHg, a large PDA with a right-to-left shunt, and improved right ventricular function compared to prior assessments, with normal left ventricular function. Blood investigations were unremarkable.

**Figure 2:**
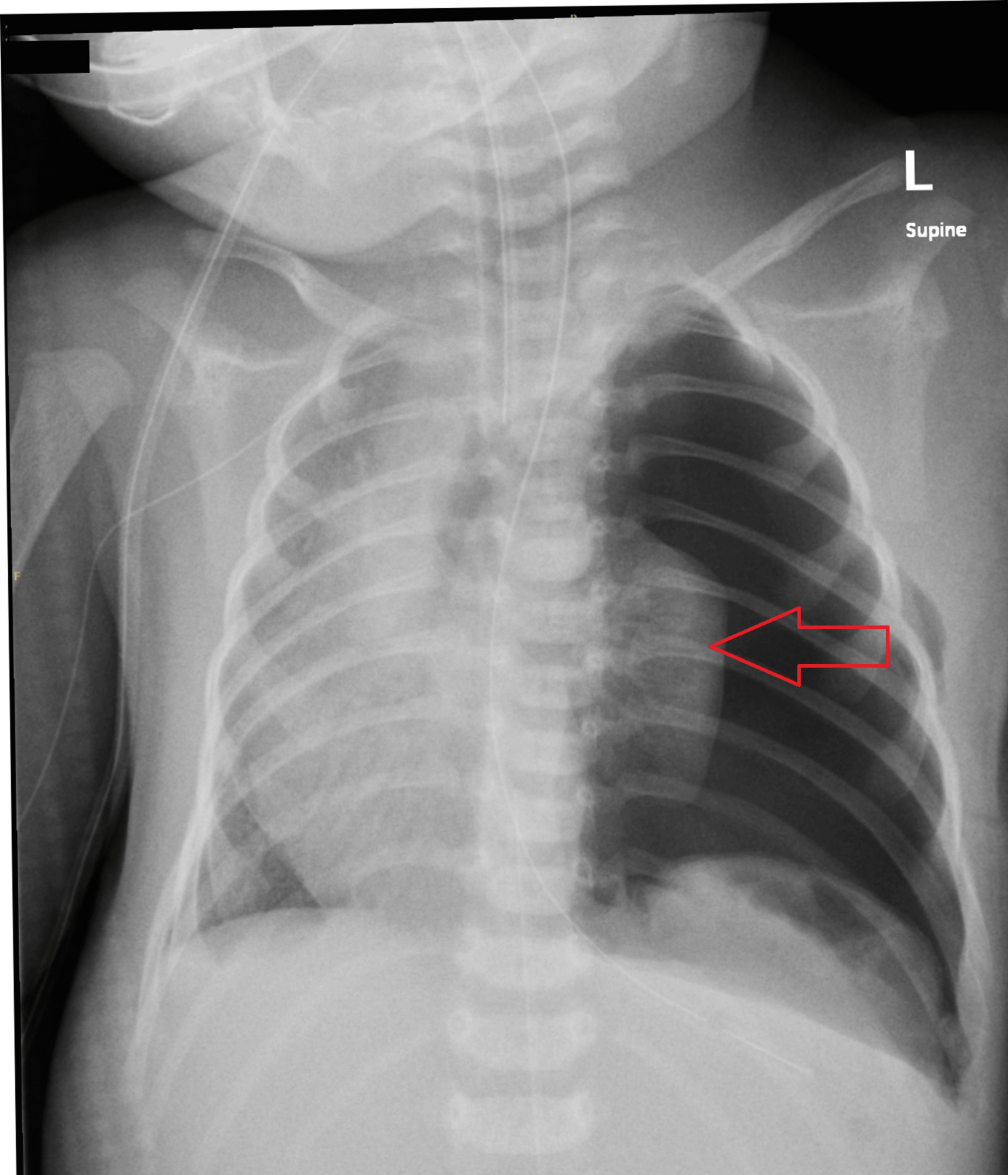
Chest X-ray performed after thoracoscopic repair of a left congenital diaphragmatic hernia revealed hypoplastic left lung (arrow).

The patient underwent a thoracoscopic repair of a left diaphragmatic hernia, a complex procedure lasting eight hours and performed in the right lateral position. Upon arrival in the operating theatre, the patient was already on infusions of milrinone at 0.25 mcg/kg/min, and vasopressin at 0.00034 units/kg/min, reflecting a meticulously coordinated preoperative management plan. Induction was expertly performed with boluses of ketamine at 1 mg/kg and midazolam at 0.1 mg/kg. Maintenance was achieved exclusively with boluses of fentanyl. Additionally, a left-sided erector spinae block was performed as an adjunct using 1 mL of 1% ropivacaine under ultrasound guidance, emphasizing a strong commitment to minimizing discomfort and optimizing analgesia. Throughout the procedure, ventilation was effectively maintained using pressure-controlled HFOV via an ICU ventilator, illustrating a deep understanding of the patient’s delicate respiratory needs. Comprehensive monitoring included ECG leads, pre-ductal SpO2 on the right wrist, post-ductal SpO2 on the right foot, and invasive blood pressure monitoring via an umbilical arterial catheter, while continuous urine output measurement remained normal at 2 mL/kg/hour. The total intravenous fluid administration of 80 mL of dextrose 5% normal saline at a rate of 12 mL/hour further emphasized meticulous fluid management. Anesthesia was maintained solely with two boluses of fentanyl, avoiding inhalational agents, which was a judicious decision given the patient’s condition. During the procedure, an initial insufflation pressure of 11 mmHg led to an elevation in PCO2 to 113 mmHg and a decrease in target ventilation. This was promptly addressed by reducing the insufflation pressure to 6-8 mmHg and transitioning to pressure control ventilation mode. Such proactive measures ensured stable hemodynamics throughout the procedure, with milrinone carefully titrated to a maximum infusion dose of 0.75 mcg/kg/min. Overall, the anesthesia management was exemplary, successfully maintaining stable blood pressure without noticeable fluctuations, highlighting the team’s diligence, expertise, and unwavering commitment to patient safety and comfort throughout this intricate surgical intervention, and chest X-ray done after the procedure as shown in Figure 2.

## Discussion

Anesthetic management of neonates with CDH requires a comprehensive approach, especially during complex procedures like the thoracoscopic repair performed on this five-day-old patient. The eight-hour surgical duration posed inherent risks, particularly due to the patient’s compromised respiratory function from left lung collapse. Continuous hemodynamic and ventilatory monitoring were crucial, as prolonged anesthesia can affect cardiovascular stability and pulmonary perfusion.

Anesthetic technique and analgesia

In this case, a left-sided erector spina block (ESB) was utilized to provide effective analgesia, significantly reducing the need for systemic opioids and inhalational anesthetics. The ESB technique involves injecting local anesthetic into the fascial plane above the erector spinae muscles, targeting the dorsal rami of spinal nerves to relieve pain in the thoracic region. This regional anesthesia approach is particularly beneficial in neonates, where minimizing opioid use is critical due to potential adverse effects. Studies demonstrate that regional techniques can enhance postoperative pain management and decrease opioid consumption, reducing the incidence of opioid-related side effects such as sedation and nausea [4].

By effectively blocking pain pathways, the ESB allowed for lower doses of opioids and decreased reliance on inhalational agents, contributing to more stable hemodynamics throughout the prolonged procedure. This not only optimized analgesia but also supported quicker recovery, demonstrating the efficacy of the left-sided erector spinae block in enhancing patient comfort and safety during surgical interventions.

Physiological monitoring and management

Surgical management of CDH presents unique anesthetic challenges, necessitating a thorough understanding of physiological implications to optimize outcomes. Maintaining a mean arterial pressure (MAP) of at least 40-50 mmHg is critical, as inadequate perfusion can compromise organ function, particularly with pulmonary hypoplasia and elevated intra-abdominal pressure often associated with CDH [5]. Continuous monitoring of pre-ductal (right arm) and post-ductal (lower extremities) oxygen saturations is essential to detect right-to-left shunting through the ductus arteriosus, with target saturations typically above 90% to prevent systemic hypoxemia [6].

Effective ventilatory strategies are vital, including HFOV with frequencies of 10-15 Hz to enhance gas exchange while minimizing ventilator-induced lung injury, low tidal volume ventilation (approximately 4-6 mL/kg) to reduce barotrauma [7], and positive end-expiratory pressure (PEEP) set at 3-5 cm H2O to recruit collapsed alveoli [8]. Regular suctioning and careful airway management are necessary to prevent obstruction and hypoventilation. Minimizing inhaled anesthetics during general anesthesia helps preserve respiratory drive and maintain hemodynamic stability, typically using sevoflurane or desflurane at low concentrations [9].

Management of pulmonary hypertension and hemodynamics

The potential for exacerbating right-to-left shunting during surgery was a critical concern, particularly due to the patient’s significant pulmonary hypertension and a large PDA. Maintaining optimal oxygen saturation and avoiding hypercarbia was essential to minimize pulmonary vasoconstriction and prevent further shunting. The administration of milrinone played a pivotal role, with gradual titration up to a maximum dose of 0.75 mcg/kg/min to improve myocardial contractility and reduce pulmonary vascular resistance [10]. Vasopressin was also used to support systemic vascular resistance, counterbalancing potential drops in blood pressure during surgical stress [11]. This dual approach facilitated hemodynamic stability while effectively managing pulmonary hypertension.

Hypercapnia presents a significant risk in neonates, particularly those with existing pulmonary hypertension. Elevated CO2 levels during surgery impacted ventilation and contributed to increased pulmonary vascular resistance, potentially worsening hemodynamic status and promoting right-to-left shunting [12]. The anesthetic team’s prompt response to rising PCO2 levels - through adjustments in ventilation strategy and insufflation pressures - was crucial in stabilizing the patient, enabling timely interventions that mitigated hypercarbia's adverse effects on pulmonary and cardiovascular function. Careful management of ventilation settings was necessary to ensure adequate oxygenation and prevent hypercapnia, which could exacerbate pulmonary hypertension and complicate right-to-left shunting.

Patient positioning

The positioning of the patient in the right lateral decubitus position was strategically significant, enhancing ventilation in the right lung while reducing perfusion to the non-functioning left lung. This position improved overall ventilation-perfusion matching but required meticulous monitoring to avoid worsening shunting through the PDA [13].

## Conclusions

The anesthetic management of neonates with CDH is a multifaceted challenge that demands meticulous planning and real-time adaptability. This case underscores the critical importance of individualized anesthetic strategies, combining multimodal analgesia and advanced ventilatory techniques to maintain hemodynamic stability and minimize respiratory compromise. The successful thoracoscopic repair of this five-day-old neonate highlights the efficacy of a collaborative, multidisciplinary approach in high-risk pediatric surgeries. By prioritizing tailored interventions and vigilant monitoring, we can enhance safety and optimize outcomes for this vulnerable population.
